# The manner of decay of genetically defective *EYS* gene transcripts in photoreceptor-directed fibroblasts derived from retinitis pigmentosa patients depends on the type of mutation

**DOI:** 10.1186/s13287-018-1016-9

**Published:** 2018-10-25

**Authors:** Yuko Seko, Masaki Iwanami, Kiyoko Miyamoto-Matsui, Shimpei Takita, Noriyuki Aoi, Akihiro Umezawa, Seishi Kato

**Affiliations:** 1 Sensory Functions Section, Research Institute, National Rehabilitation Center for Persons with Disabilities, 4-1 Namiki, Tokorozawa, 359-8555 Japan; 2 Department of Ophthalmology, Hospital, National Rehabilitation Center for Persons with Disabilities, 4-1 Namiki, Tokorozawa, 359-8555 Japan; 3 Department of Plastic, Oral and Maxillofacial Surgery, Teikyo University School of Medicine, 2-11-1 Kaga Itabashi-ku, Itabashi, 173-8605 Japan; 4 Department of Reproductive Biology, Center for Regenerative Medicine, National Institute for Child Health and Development, 2-10-1 Okura, Setagaya, 157-8535 Japan; 5 Research Institute, National Rehabilitation Center for Persons with Disabilities, 4-1 Namiki, Tokorozawa, 359-8555 Japan; 6 Present Address: Iwanami Eye Clinic, 7-1-3, Tsuchihashi, Miyamae-ku Kawasaki-shi, Kanagawa 216-0005 Japan

**Keywords:** Redirect differentiation, dermal fibroblast, photoreceptor, disease modeling, retinitis pigmentosa, *EYS*, truncating mutation, nonsense-mediated mRNA decay (NMD), phenotypic variation, genotype-phenotype relationship

## Abstract

**Background:**

Generation of induced photoreceptors holds promise for *in vitro* modeling of intractable retinal diseases. Retinitis pigmentosa is an inherited retinal dystrophy that leads to visual impairment. The *EYS* gene was reported to be the most common gene responsible for autosomal recessive retinitis pigmentosa (arRP). arRP with defects in the *EYS* gene is denoted by “EYS-RP”. We previously established a “redirect differentiation” method to generate photosensitive photoreceptor-like cells from commercially available human dermal fibroblasts. In this study, we produced photoreceptor-like cells from dermal fibroblasts of EYS-RP patients as a replacement for the degenerative retinas using “redirect differentiation”. We analyzed defective transcripts of the *EYS* gene in these cells to elucidate phenotypes of EYS-RP patients because decay of transcripts was previously suggested to be involved in phenotypic variation associated with diseases.

**Methods:**

Using “redirect differentiation” by *CRX*, *RAX*, *NeuroD* and *OTX2*, we made photoreceptor-directed fibroblasts derived from three normal volunteers and three EYS-RP patients with homozygous or heterozygous mutations. We tested inducible expression of the photoreceptor-specific genes (blue opsin, rhodopsin, recoverin, S-antigen, PDE6C) in these cells. We then analyzed transcripts derived from three different types of the defective *EYS* gene, c.1211dupA, c.4957dupA and c.8805C > A, expressed in these cells by RT-PCR and sequencing.

**Results:**

Photoreceptor-specific genes including the *EYS* gene were up-regulated in all the photoreceptor-directed fibroblasts tested. However, expression levels of defective transcripts were markedly different depending on the type of mutation. Transcripts derived from these three defective genes were scarcely detected, expressed at a lower level, and expressed at almost the same level as in normal volunteers, respectively.

**Conclusions:**

Expression levels of genetically defective *EYS* gene transcripts in photoreceptor-directed fibroblasts of EYS-RP patients vary depending on the type of mutation. Variation in expression levels in transcripts having c.1211dupA, c.4957dupA and c.8805C > A suggests that almost complete nonsense-mediated mRNA decay (NMD), partial NMD and escape from NMD occurred for these transcripts, respectively. To determine the relationship with phenotypic variations in EYS-RP patients, more samples are needed. The present study also suggests that the redirect differentiation method could be a valuable tool for disease modeling despite some limitations.

**Electronic supplementary material:**

The online version of this article (10.1186/s13287-018-1016-9) contains supplementary material, which is available to authorized users.

## Background

Retinitis pigmentosa (RP) is an inherited retinal dystrophy that leads to visual loss. Defects in the *EYS* gene on chromosome 6q12 were found to be a major cause of autosomal recessive (ar) retinitis pigmentosa (RP) in several countries [1–6]. In Japan, c.4957dupA (p.Ser1653Lysfs*2) and c.8805C > A (p.Tyr2935*) were identified as pathogenic mutations detected in about 20%〜30% of arRP patients [7, 8]. Hereafter arRP caused by defects in the *EYS* gene is denoted by “EYS-RP”. Differences in long-term prognoses of EYS-RP patients with homozygous and heterozygous mutations have previously been suggested [7]. Additionally, other types of mutations in the *EYS* gene have been reported. RP is a highly heterogeneous disease, and accordingly, EYS-RP exhibits heterogeneous phenotypes with a wide range in severity. In order to clarify the genotype-phenotype correlation in EYS-RP, analysis of transcripts may be helpful.

*EYS* was reported as the human ortholog of the *Drosophila*, eye shut (*eys*) gene [1, 4]. The *eys* was identified as an agrin/perlecan-related extracellular matrix protein and is secreted by photoreceptors into the interrhabdomeral space, playing an essential role in the formation of epithelial lumina in the fly retina [9]. The lumen was thought to be compatible to subretinal space of the human retina, which plays an important function in vision. *EYS* (OMIM 612424) is the largest gene currently known to be expressed in the human eye, spanning over 2 Mb within the RP25 locus (6q12) [1, 4]. The ideal tool for analysis of the *EYS* gene transcripts is a retina from the EYS-RP patient. For research purposes, cellular models are available as a replacement for the human retinas. Induced photoreceptor cells generated from disease-specific iPSCs of RP patients were reported to reproduce pathogenic phenotypes [10–14]. Although methods to generate photoreceptors from iPSCs have been established [15, 16], they are expensive and time-consuming. We established another method called “redirect differentiation”, by which photosensitive photoreceptor-like cells are generated more easily and rapidly [17–19].

In this study, using “redirect differentiation”, we produced photoreceptor-like cells (photoreceptor-directed fibroblasts) from dermal fibroblasts of EYS-RP patients with homozygous or heterozygous mutations, as a replacement for the degenerative retinas from EYS-RP patients. It has been reported that decay of transcripts might be a possible determinant of phenotypic variation [20]. And it was reported that nonsense mediated mRNA decay (NMD) plays a role in phenotypic differences found in diseases [21] and controls of diseases [22]. Together with gene transcription, cytoplasmic mRNA decay plays an important role in regulating the level of protein-encoding gene expression, mainly through AU-rich element-mediated control [23]. On the other hand, in order to maintain the quality of gene expression, eukaryotes have mechanisms such as nonsense-mediated decay (NMD), nonstop decay (NSD), no-go decay (NGD) and ribosome extension-mediated decay (REMD) [24]. In the present study, transcripts from 3 EYS-RP patients with truncating mutations were investigated for NMD. In order to investigate the decay of abnormal *EYS* gene transcripts in degenerated retinas, we here analyzed the defective transcripts of the *EYS* gene in differentiated cells as a cellular model of EYS-RP. Because it has been reported that most pathogenic mutations are truncating ones [2, 3, 5–8, 25], three patients with truncating mutations (Table 1) were recruited. Pt#1 was known as RP38 in our previous paper [7] and Pt#2 and Pt#3 were new patients.

**Table 1:** Defects in the *EYS* gene in patients for this study

Patient #	ID	Sex	Age (y)	Allele 1		Allele 2	
				Mutation	Effect	Mutation	Effect
1	RP38	F	67	c.4957dupA	p.Ser1653Lysfs*2	c.4957dupA	p.Ser1653Lysfs*2
2	RP174	M	58	c.4957dupA	p.Ser1653Lysfs*2	c.8805C > A	p.Tyr2935*
3	RP165	F	79	c.4957dupA	p.Ser1653Lysfs*2	c.1211dupA	p.Asn404Lysfs*3

We here demonstrate that manners of decay of the *EYS* gene transcripts found in photoreceptor-directed fibroblasts derived from EYS-RP patients vary depending on defect variations of the *EYS* gene in the genome. Our results could be helpful in clarifying the genotype-phenotype relationship in EYS-RP patients. The present study also suggests that the redirect differentiation method could be a useful and valuable tool for disease modeling despite its limitations.

## Methods

### Isolation and culture of dermal fibroblasts

Dermal fibroblasts were harvested from three healthy donors (a 53 year-old male (N#1), a 48 year-old male (N#2), a 63 year-old male (N#3)) and three EYS-RP patients (Table 1) under the approval of the Ethics Committee of the National Rehabilitation Center for Persons with Disabilities (NRCD). Signed informed consent was obtained from the six donors and samples were de-identified. All experiments involving human cells and tissues were performed in line with the Declaration of Helsinki. The skin of N#1 was excised from two different areas. One was excised in a circle-shape by a trephine (2 mm diameter) from the periauricular area and another was excised from the flexion side of the elbow by the same type of trephine. The skin of other subjects, N#2, N#3 and three RP patients, was excised from only the elbow. The dermises were separated from the skin and attached to scratched 35 mm dishes. Explanted dermises were then cultured in Dulbecco’s modified Eagle’s medium (DMEM) with 10% fetal bovine serum (Hyclone) for two weeks until human dermal fibroblasts migrated and proliferated around the explant dermises. Fibroblasts from the outgrowth areas were passaged 3 or 4 times and then frozen in aliquots. Seventh-passaged cells were used for following photoreceptor-directed differentiation. We investigated the growth of dermal fibroblasts of N#1, N#3, Pt#1 and Pt#2. The number of cells increased until about 14 days; however, growth rates were different depending on the donor (Additional file 1: Figure S1).

### Induction of photoreceptor-like cells

Induction experiments were performed as previously reported [17, 18]. In brief, full-length transcription factors, *OTX2* [26], *RAX* [27], *CRX* [28], and *NeuroD* [29], were amplified from cDNAs prepared from total RNA of adult human retina (Clontech, CA, USA) by PCR, and cloned into the XmnI-EcoRV sites of pENTR11 (Invitrogen). Preparation and infection of recombinant retrovirus were performed as previously reported [17]. In brief, the resulting pENTR11-transcription factors were recombined with pMXs-DEST by use of LR recombination reaction as instructed by the manufacturer (Invitrogen). The retroviral DNAs were then transfected into 293FT cells and 3 days later the media were collected and concentrated. The human dermal fibroblasts were infected with this media containing retroviral vector particles as a mixture of four kinds of transcription factors. After the retroviral infection, the media were replaced with the DMEM/F12/B27 medium supplemented with 40 ng/ml bFGF, 20 ng/ml EGF, fibronectin, and 1% FBS. The retrovirus-infected cells were cultured for up to 10–21 days. We transduced retroviral eGFP under the same condition to measure efficiency of infection. The frequency of eGFP-positive cells was 90–94% of all cells at 48 h after infection. Expression of 4 transgenes (*CRX, RAX, NeuroD* and *OTX2*) was also confirmed by endpoint RT-PCR in photoreceptor-directed fibroblasts derived from all cells types tested (Additional file 1: Figure S2). By immunocytochemistry, we found cells positively stained for blue opsin and CRX in all photoreceptor-directed fibroblasts tested (Additional file 1: Figure S3).

### Reverse transcriptase-PCR

Total RNA was isolated with an RNeasy Plus mini kit® (Qiagen, Maryland, USA) or PicoPure™ RNA Isolation Kit (Arcturus Bioscience, CA, USA) according to the manufacturer’s instruction. An aliquot of total RNA was reverse transcribed by using an oligo (dT) primer. The design of PCR primer sets for the photoreceptor-specific genes were shown in our previous paper [17] and those for the *EYS* gene in the Additional file 1: Table S1 for this paper. The nucleotide sequence of the *EYS* gene (NM_001142800.1) was retrieved from the National Center for Biotechnology Information (NCBI). Nucleotide A of the initiation codon of *EYS* was defined as position 1.

### Quantitative RT-PCR

A cDNA template was amplified using the Platinum Quantitative PCR SuperMix-UDG with ROX (Invitrogen) and ABI7900HT Sequence Detection System (Applied Biosystems). Fluorescence was monitored during every PCR cycle at the annealing step. The authenticity and size of the PCR products were confirmed using a melting curve analysis (using software provided by Applied Biosystems) and a gel analysis. An mRNA level was normalized using G3PDH as a housekeeping gene. The design of PCR primer sets is shown in our previous paper [17] and Additional file 1: Table S1.

### Sequencing

Direct sequencing was performed with a BigDye® Terminator Cycle Sequencing Kit (Applied Biosystems, Foster City, CA). Sequencing reaction products were run on an automated capillary sequencer (3130xl Genetic Analyzer; Applied Biosystems).

### Cell counting

Fibroblasts grown in 12-well culture dishes were counted with TC10™ automated cell counter (Bio-Rad). The number of total cells without trypan blue staining was used. Seventh-passaged dermal fibroblasts derived from N#1, N#3, Pt#1 and Pt#2 were seeded at 1.5 × 10^4^ cells per one well of the 12–well culture dish on day 0. After cultivation for different period of time, adherent cells were trypsinized and counted.

### Immunocytochemistry

Immunocytochemical analysis was performed as previously described [17, 18]. As a methodological control, the primary antibody was omitted. The primary antibodies used were as follows: blue opsin (1: 50, goat polyclonal, P-13, Santa Cruz, CA, USA), CRX (1: 50, rabbit polyclonal, H-120, Santa Cruz, CA, USA). 0.5 × 10^5^ cells were seeded on laminin-coated glass-bottomed 35 mm culture dishes. Fourteen days after induction, photoreceptor-directed fibroblasts were prepared for immunocytochemistry. Cells were fixed with 4% PFA in PBS for 20 min. Cells were rinsed in PBS and incubated overnight with primary antibodies described above. After washing, cells were incubated with secondary antibodies (1:500; Life Technologies, CA, USA). Nuclei were stained with DAPI. Cells were mounted using fluorescence mounting medium (Dako, Tokyo, Japan). Detection of digital images was performed by a Nikon ECLIPSE TE300 and NIS-Elements AR3.0 software (Nikon, Tokyo, Japan).

## Results

### Photoreceptor-directed fibroblasts express the *EYS* gene within 10 days after transduction of retina-related transcription factors via retroviral vectors

**Fig. 1:**
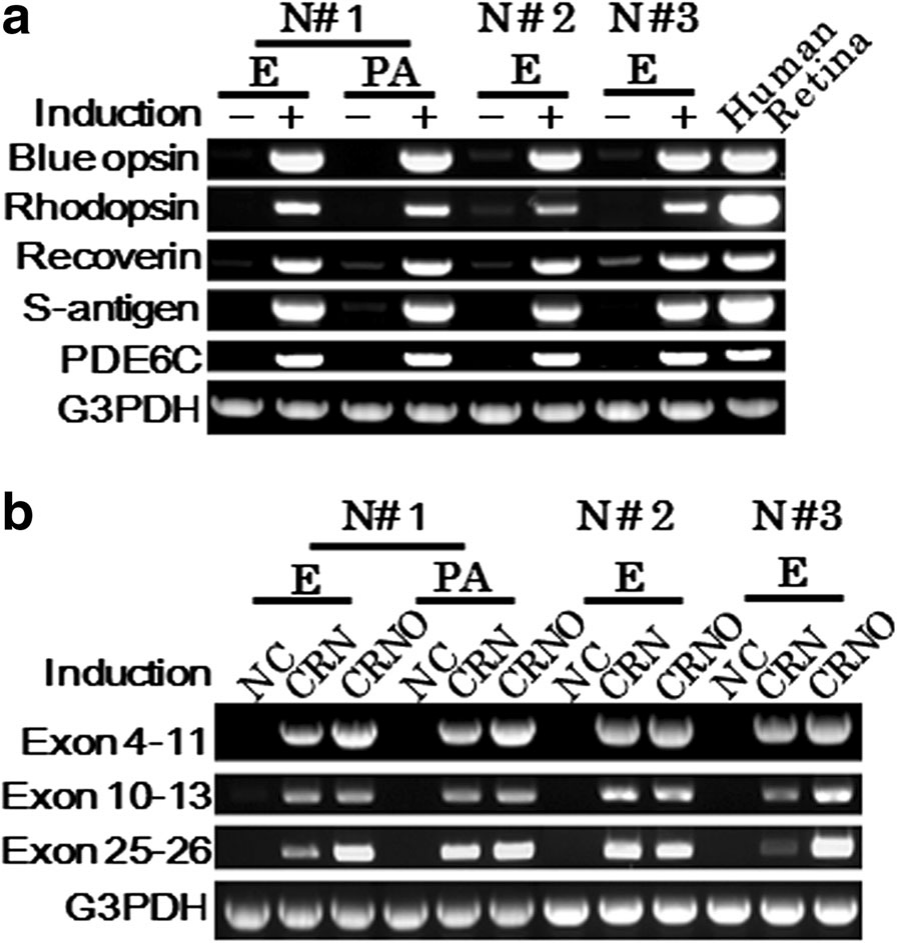
Induction of retina-specific genes in human dermal fibroblasts by the retroviral infection of genes for defined transcription factors. **a** RT-PCR analysis for photoreceptor-specific genes. In human dermal fibroblasts of three normal volunteers (N#1, N#2, N#3), blue opsin, rhodopsin, recoverin, S-antigen and PDE6C genes were induced by *CRX*, *RAX*, *NeuroD* and *OTX2* transduction 10 days after gene transduction. Skin was excised from two different areas. One (PA) was excised from the periauricular area and another (E) was excised from the elbow. “Human retina”: a human retinal tissue as a positive control. **b** RT-PCR analysis for the *EYS* gene. The regions for exon 4–11, exon 10–13, and exon 25–26 of the *EYS* gene were amplified. The *EYS* genes were expressed in dermal fibroblasts derived from both the periauricular area and the elbow 10 days after gene transduction. “NC”: fibroblasts without gene transduction, “CRN”: *CRX*, *RAX* and *NeuroD* transduction, “CRNO”: *CRX*, *RAX*, *NeuroD* and *OTX2* transduction

In our previous paper, we showed that commercially available dermal fibroblast cell lines infected with four transcription factors, *CRX, RAX, NeuroD* and *OTX2*, express several photoreceptor-specific genes [18]. In the present study, human dermal fibroblasts derived from three normal volunteers (N#1, N#2, N#3) were examined for inducible expression of several photoreceptor-related genes. RT-PCR results showed that transduction of these four genes up-regulated the expression of the photoreceptor-specific genes (blue opsin, rhodopsin, recoverin, S-antigen, PDE6C) in four types of fibroblasts tested (Fig. 1, Panel **a**). The expression of *EYS* genes was then investigated. Because of the long size of the *EYS* gene transcript, three different primer pairs were designed and tested. The amplified products corresponding to each primer pair were clearly detected (Fig. 1, Panel **b**) and confirmed by direct sequencing to be the *EYS* gene transcripts. Cells used for RT-PCR were collected 10 days after gene transduction. Photoreceptor-specific genes including the *EYS* gene were up-regulated in dermal fibroblasts derived from both periauricular area and the flexion side of the elbow in N#1. Therefore, we collected fibroblasts from only the elbow because of convenience.

### Detection of *EYS* transcripts in photoreceptor-directed fibroblasts from EYS-RP patients

In our previous studies using iris cells, expression levels of rhodopsin and blue opsin reached a maximum level one week after gene transduction and remained unchanged for up to 3 weeks [17]. In the present study, photoreceptor-directed fibroblasts from 3 EYS-RP patients tested 10 days after gene transduction exhibited gene expression of blue opsin and S-antigen as detected by endpoint RT-PCR (Additional file 1: Figure S4). We then analyzed the expression level of the *EYS* gene during photoreceptor-directed differentiation using primer pairs for exon 8–9. As a result, the expression levels of the *EYS* gene in Pt#1, Pt#2 and N#2 reached maximum levels around 10 to 14 days after gene transduction and remained unchanged for up to 3 weeks (Additional file 1: Figure S5, Panel **a**). Then, using total RNAs extracted from photoreceptor-directed fibroblasts of Pt#1, Pt#2 and Pt#3 10 days after gene transduction, we performed RT-PCR and analyzed DNA sequences of amplified products for exon 26–27, exon 42–43 and exon 6–11 that carry c.4957dupA, c.8805C > A and c.1211dupA, respectively.

**Fig. 2:**
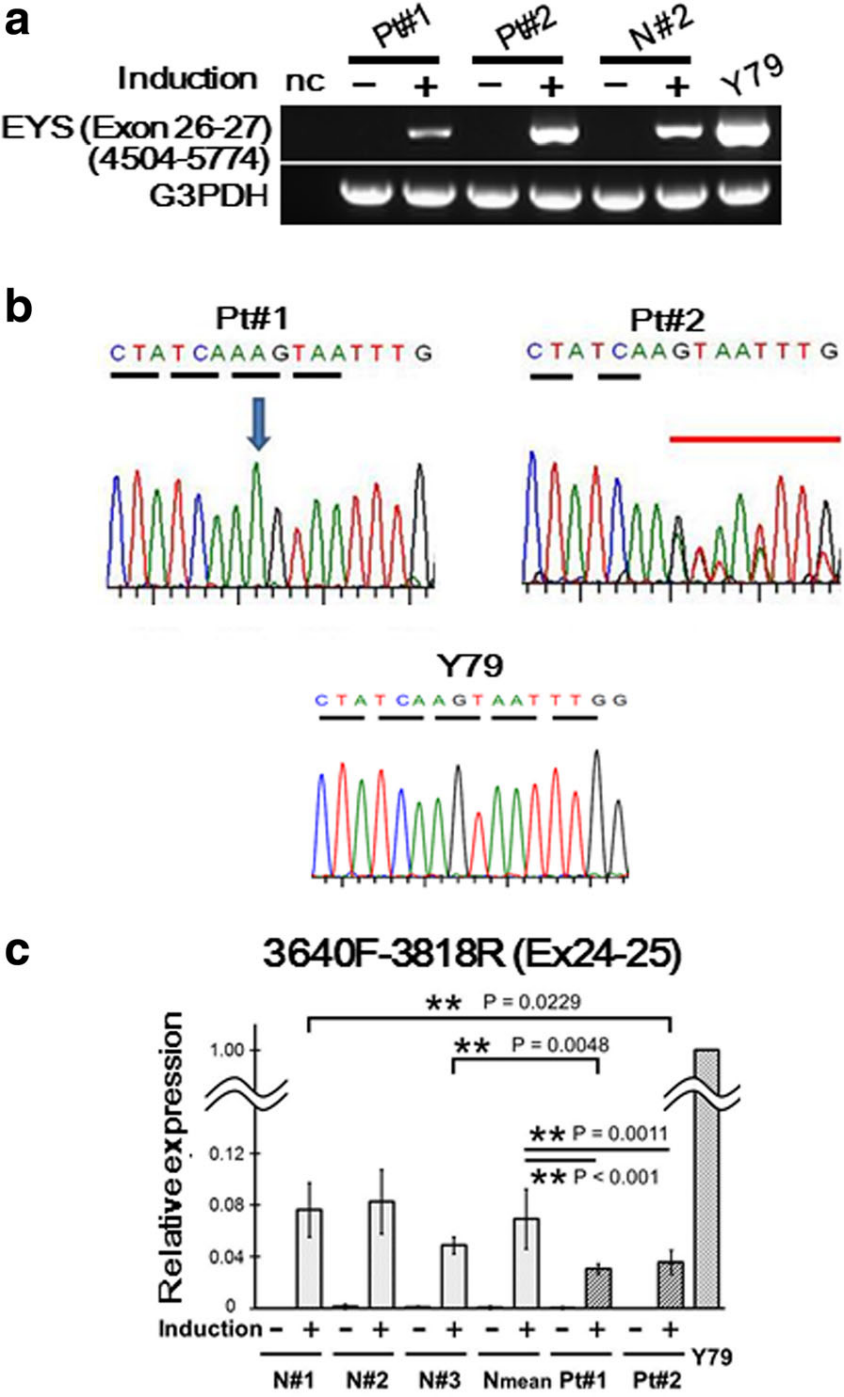
Detection of expression and identification of sequences of *EYS* transcripts corresponding to exon 26–27 that includes c.4957dupA in photoreceptor-directed fibroblasts from EYS-RP patients. **a** RT-PCR analysis of expression of *EYS* gene using primer pairs corresponding to exon 26–27. Expression levels of *EYS* gene were clearly up-regulated by *CRX*, *RAX, NeuroD* and *OTX2* transduction in Pt#1, Pt#2 and N#2 10 days after transduction. Y79 was used as a positive control. **b** Electropherogram corresponding to c.4957dupA by sequencing of RT-PCR products. The transcripts had c.4957dupA mutation in photoreceptor-directed fibroblasts from Pt#1carrying homozygous mutation and Pt#2 carrying compound heterozygous mutation. As for Pt#2, the peak amplitudes of mutated bases on the electropherogram seemed to be lower than normal bases. **c** Analysis of expression levels of *EYS* gene corresponding to exon 24–25 by qRT-PCR. Vertical axis indicates relative expression (mean ± SD, *n* = 4 for Pt#1 and Pt#2, *n* = 3 for N#1, N#2 and N#3). To compare expression levels of defected *EYS* gene transcripts (Pt#1 and Pt#2) to normal (N#1, N#2, N#3), we performed qRT-PCR for photoreceptor-directed fibroblasts 10 days after transduction. We designed primer pairs nearly upstream to c.4957dupA (exon 24–25, 3640F-3818R) and compared the expression levels of *EYS* gene corresponding to exon 24–25. Expression levels of exon 24–25 were significantly lower in Pt#1 (Wilcoxon test, *p* < 0.001) and Pt#2 (*P* = 0.0011) compared to the average of three normal volunteers. Those of Pt#1 was also lower than age-matched control, N#3 ((Wilcoxon test, p < 0.001)

Generally, faulty transcripts are triaged for destruction by NMD immediately [30]. All three EYS-RP donors have the frameshift mutation, c.4957dupA, in at least one allele of exon 26 (Table 1). Therefore, we expected that NMD would cause the disappearance of the *EYS* gene transcripts corresponding to exon 26–27. However, the transcripts having c.4957dupA were clearly detected in photoreceptor-directed fibroblasts from Pt#1carrying homozygous mutations and Pt#2 carrying compound heterozygous mutations (Fig. 2, Panels **a** and **b**). However, the expression levels in Pt#1 and Pt#2 were lower than the mean of N#1, N#2 and N#3 (Fig. 2, Panel **c**). As for Pt#2, the peak amplitudes of mutated bases on the electropherogram seemed to be lower than normal bases (Fig. 2, Panel **b**), although quantitative estimation of expression levels based on elecropherogram may be limited because of 30 cycles of amplification in the cycle sequencing protocol used here.

**Fig. 3:**
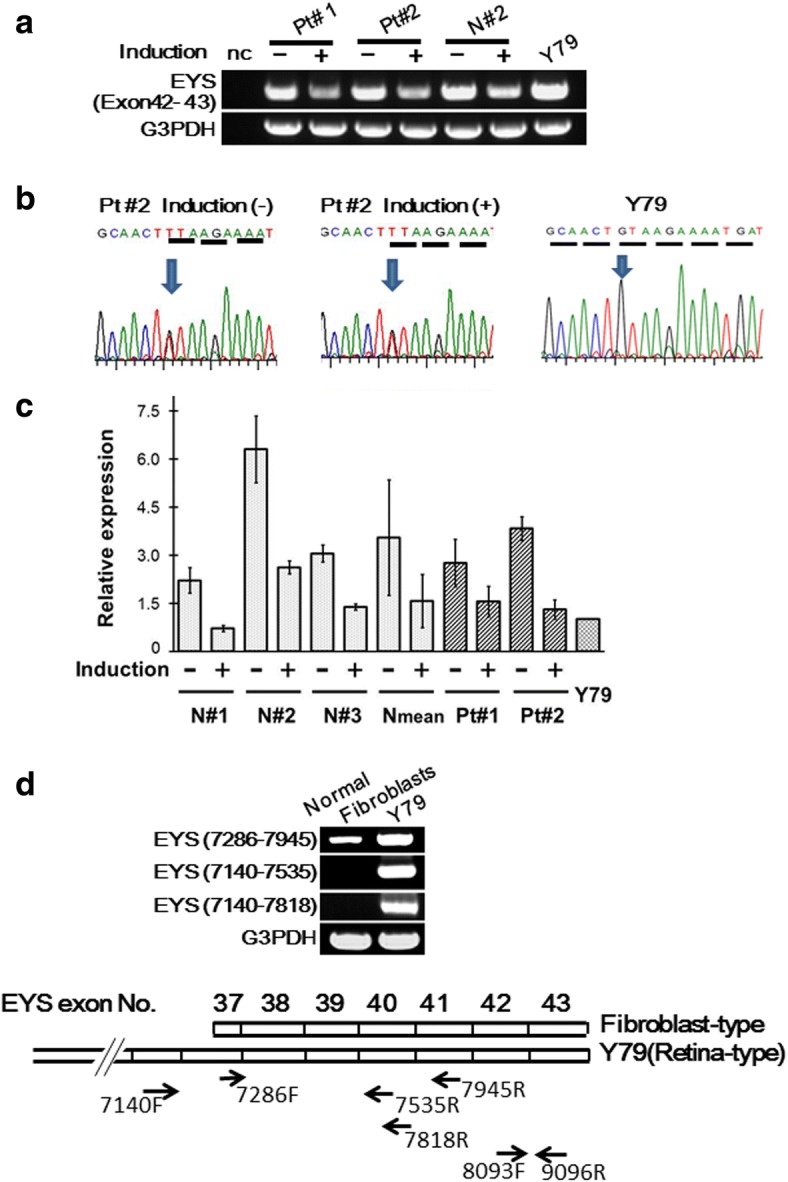
Detection of expression and identification of sequences of *EYS* gene transcripts corresponding to exon 42–43 that includes c.8805C > A in photoreceptor-directed fibroblasts from EYS-RP patients. **a** RT-PCR analysis of expression of *EYS* gene using primer pairs corresponding to exon 42–43. Expression levels of the *EYS* gene were clearly down-regulated by *CRX*, *RAX, NeuroD* and *OTX2* transduction in Pt#1, Pt#2 and N#2 10 days after transduction. Y79 was used as a positive control. Interestingly, the exon 42–43 region of the *EYS* gene was expressed in human dermal fibroblasts without photoreceptor-induction. The expression level of the exon 42–43 fragment in default state fibroblasts was higher than in photoreceptor-directed fibroblasts. **b** Electropherogram corresponding to c.8805C > A by sequencing of RT-PCR products. The nucleotide sequence in this panel shows a complementary strand. The transcripts having c.8805C > A mutation were found both in photoreceptor-directed fibroblasts and default state fibroblasts from Pt #2. The peak amplitudes of normal and mutated bases on the electropherogram were nearly the same. **c** Analysis of expression levels of the *EYS* gene corresponding to exon 42–43. Vertical axis indicates relative expression (mean ± SD, n = 4 for Pt#1 and Pt#2, n = 3 for N#1, N#2 and N#3). The expression levels of the exon 42–43 fragment in default state fibroblasts were higher than in photoreceptor-directed fibroblasts 10 days after transduction. **d** Analysis of expression of the *EYS* gene in the default state fibroblasts by endpoint RT-PCR corresponding to exon 36–41. RT-PCR products amplified with the forward primer designed at exon 36 (EYS 7140) were detected only in Y79. On the other hand, RT-PCR products amplified with the forward primer designed at exon 37 (EYS 7286) were detected both in normal fibroblasts and Y79. These results suggest that the transcription start site of fibroblast-type exists between EYS 7140 (exon 36) and EYS 7286 (exon 37). 8093F and 9096R correspond to the forward and reverse primers used for the exon 42–43 fragments

Pt#2 has the nonsense mutation, c.8805C > A, on an allele of exon 43 (Table 1). Because this mutation produces a premature termination codon (PTC) in the last exon, the transcript with this mutation may escape from degradation by NMD [21]. In the present study, these transcripts corresponding to exon 42–43 were clearly detected in photoreceptor-directed dermal fibroblasts derived from Pt#2 (Fig. 3, Panel **a**). Because the amplified product had the mutation, c.8805C > A, one of the transcripts was confirmed to be derived from the mutated allele (Fig. 3, Panel **b**). The peak amplitudes of normal and mutated bases on the electropherogram were nearly the same. The expression levels from Pt#2 were similar to those from normal volunteers (Fig. 3, Panel **c**).

Interestingly, the exon 42–43 region of the *EYS* gene was expressed in human dermal fibroblasts without photoreceptor-induction. The expression level of the exon 42–43 fragment in default state fibroblasts was higher than in photoreceptor-directed fibroblasts (Fig. 3, Panels **a** and **c**).

**Fig. 4:**
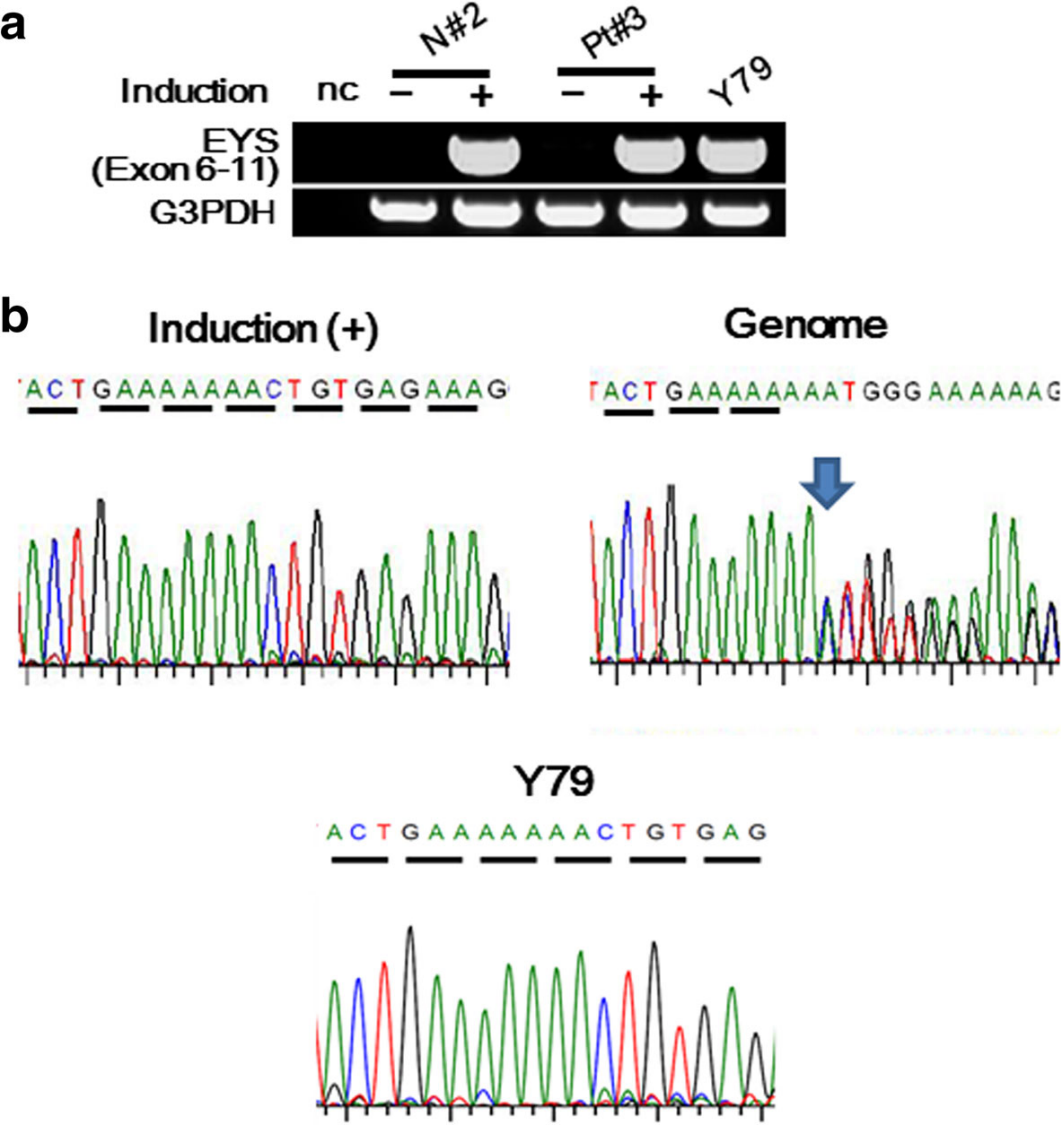
Detection of expression and identification of sequences of *EYS* gene transcripts corresponding to exon 6–11 that includes c.1211dupA in photoreceptor-directed fibroblasts from EYS-RP patients. **a** RT-PCR analysis of expression of the *EYS* gene using primer pairs corresponding to exon 6–11. Expression levels of *EYS* gene were clearly up-regulated by *CRX*, *RAX, NeuroD* and *OTX2* transduction in Pt#3 and N#2 10 days after transduction. Y79 was used as a positive control. **b** Electropherogram corresponding to c.1211dupA by sequencing of RT-PCR products and the genome. Sequencing analyses revealed that these RT-PCR products corresponding to exon 6–11 consisted of normal sequences with an extremely low level of defected sequences

Pt#3 has the frameshift mutation, c.1211dupA (p.Asn404Lysfs*3) in exon 8, that has previously been reported in an Israeli arRP patient [5]. By endpoint RT-PCR and sequencing, only transcript derived from the normal allele was detected (Fig. 4), suggesting that transcript derived from the mutant allele was degraded by NMD as expected.

### Compositional changes of *EYS* gene transcripts over time during photoreceptor-directed differentiation

**Fig. 5:**
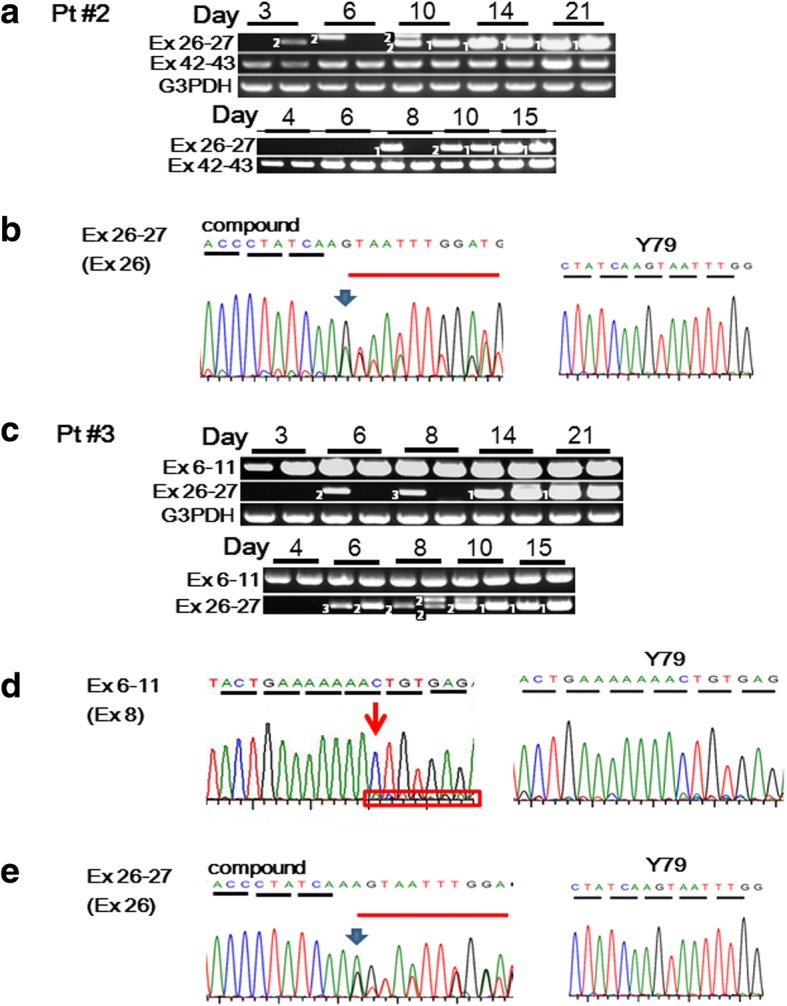
Changes of compositions of *EYS* gene transcripts over time during photoreceptor-directed differentiation. **a** Changes of compositions of *EYS* gene transcripts of Pt#2 by endpoint RT-PCR and sequencing. **b** Representative patterns of electropherogram corresponding to exon 26–27 including c.4957dupA. Endpoint RT-PCR products amplified for exon 26–27 and exon 42–43 at 3, 4, 6, 8, 10, 14, 15 and 21 days during photoreceptor-directed differentiation for Pt#2 (a). Two independent induction-experiments were performed for 3–21 days and for 4–15 days. In each time-course experiment, induction was performed in duplicate and endpoint RT-PCR was performed using each induced sample. Sequencing analyses revealed that these RT-PCR products corresponding to exon 26–27 derived from Pt#2 consisted of either compound transcripts of normal and mutated sequences (1) or only mutated sequence (2) at some time points before 14 days. After 14 days, amplified fragments contained compound transcripts of normal and mutated sequences. Representative electropherogram of “compound” pattern shown in panel b indicates the peak amplitudes of mutated bases were lower than normal bases. Sequences of RT-PCR products corresponding to exon 42–43 for Pt#2 showed compound transcripts of normal and mutated sequences at all the time points during induction. Endpoint RT-PCR products corresponding to exon 26–27 including c.4957dupA, yielded products with different mobility before 10 days (a). Sequencing the PCR products verified that these were alternatively spliced *EYS* gene transcripts (Additional file 1: Figure S6). **c** Changes of compositions of *EYS* gene transcripts of Pt#3 by endpoint RT-PCR and sequencing. **d**, **e** Representative patterns of electropherogram corresponding to exon 6–11 including c.1211dupA (d) and corresponding to exon 26–27 including c.4957dupA (e). Endpoint RT-PCR products amplified for exon 6-11and exon 26–27 at 3, 4, 6, 8, 10, 14, 15 and 21 days during photoreceptor-directed differentiation for Pt#3 (c). Two independent induction-experiments were performed for 3–21 days and for 4–15 days. In each time-course experiment, induction was performed in duplicate and endpoint RT-PCR was performed using each induced sample. Sequencing analyses revealed that these RT-PCR products corresponding to exon 6–11 consisted of normal sequences with a very low level of defected sequences (d). Sequencing analyses revealed that these RT-PCR products corresponding to exon 26–27 consisted of either compound transcripts of normal and mutated sequences ((1), “compound” (e)), only mutated sequence (2) or only normal sequence (3) at some time points before 14 days. However, after 14 days, amplified fragments contained compound transcripts of normal and mutated sequences. The electropherogram (e) indicates the peak amplitudes of normal bases were lower than mutated bases

Pt#2 and Pt#3 have compound heterozygous mutations for c.4957dupA. We performed endpoint RT-PCR and analyzed DNA sequences of RT-PCR products amplified for exon 26–27, exon 42–43 and exon 6–11 using abovementioned primers at 3, 4, 6, 8, 10, 14, 15 and 21 days during photoreceptor-directed differentiation. Sequencing analyses revealed that these RT-PCR products corresponding to exon 26–27 derived from Pt#2 consisted of either compound transcripts of normal and mutated sequences (1) or only mutated sequence (2) at some time points before 14 days (Fig. 5, Panel **a**). Those from Pt#3 consisted of either only mutated sequence (2) or compound transcripts of normal and mutated sequences (1) or only normal sequence (3) at some time points before 14 days (Fig. 5, Panel **c**). However, after 14 days, all amplified fragments contained compound transcripts of normal and mutated sequences (Fig. 5, Panel **b**). Sequences of RT-PCR products corresponding to exon 42–43 for Pt#2 showed compound transcripts of normal and mutated sequences at all the time points during induction. Sequencing analyses revealed that these RT-PCR products corresponding to exon 6–11 derived from Pt#3 consisted of normal sequences with a very low level of defected sequences (Fig. 5, Panel **d**).

Before 10 days, RT-PCR with the primer pairs for exon 26–27 yielded products with different mobility (Fig. 5, Panels **a** and **c**). Sequencing the PCR products verified that these were alternatively spliced *EYS* gene transcripts containing a novel exon between exon 26 and 27 (Additional file 1: Figure S6). It remains to be determined whether these transcripts are related to photoreceptor-directed differentiation or EYS-RP pathogenesis.

## Discussion

This study is the first to analyze retina-specific *EYS* gene transcripts in photoreceptor-directed fibroblasts derived from EYS-RP patients as cellular models. As a result, transcripts derived from mutated alleles, c.1211dupA, c.4957dupA and c.8805C > A, were barely detected, expressed at a lower level, and expressed at almost the same level as in normal volunteers, respectively. These variations in expression levels suggest that almost complete NMD, partial NMD and escape from NMD occurred, respectively. These results suggest that the mechanism of decay of genetically defective *EYS* gene transcripts in photoreceptor-directed fibroblasts varies depending on defect variations of the *EYS* gene in the genome.

In a previous paper, we showed that 32.8% of arRP patients have the frameshift mutation, c.4957dupA, and/or the nonsense mutation, c.8805C > A, as pathogenic mutations [7]. These mutations produce PTCs in a transcript, leading to NMD or production of truncated proteins. In order to clarify the fate of the transcripts with these PTCs, the ideal strategy is analysis of the *EYS* gene transcripts in a retina from the EYS-RP patient. However, because a sample of the retina is not available, we applied our method of redirecting differentiation into photoreceptor-like cells [18] for analysis of the *EYS* gene transcripts in the EYS-RP patient. We here found that photoreceptor-like cells from dermal fibroblasts express *EYS* gene transcripts, suggesting that further analysis of defective *EYS* gene transcripts of EYS-RP patients should be possible by using these cells. We then produced photoreceptor-like cells from dermal fibroblasts derived from three EYS-RP patients, Pt#1carrying homozygous mutations (c.4957dupA), Pt#2 carrying compound heterozygous mutations (c.4957dupA and c.8805C > A) and Pt#3 carrying compound heterozygous mutations (c.1211dupA and c.4957dupA). We performed semi-quantitative analyses of *EYS* gene transcripts expressed in cellular models of degenerated retinas of EYS-RP patients, and estimated the extent of NMD from the expression levels of defective *EYS* gene transcripts.

All three patients tested here have c.4957dupA as a homozygous or heterozygous mutation (Table 1). The transcripts having c.4957dupA were clearly detected in photoreceptor-directed fibroblasts from all three patients. However, the expression levels of the *EYS* gene fragment corresponding to exon 26–27 in Pt#1 were lower than those from normal volunteers (Fig. 2), suggesting that transcripts with c.4957dupA may be degraded by NMD to some extents but may escape from NMD in part. It is believed in general that faulty transcripts are immediately triaged for destruction by NMD [30]. Several *trans*-acting factors were identified as potential mechanisms [22, 31, 32]. Additionally, mechanisms involving *cis*-acting factors have predicted that a PTC resides ≧ 50–55 nt upstream of an exon-exon junction stimulates NMD [33] and that the length of the 3’UTR is an important factor that influences NMD [34–36]. Recently, it was also reported that a *cis* element is located within the first 200 nt that inhibits NMD when positioned in downstream proximal region of the PTC [37]. They showed several examples with a significant enrichment of A/U nucleotides (63%–71%) and hypothesized that this may be a condition for NMD evasion. To determine whether our data support their hypothesis, we analyzed sequences in the proximal downstream region of the PTC in the transcripts derived from defected alleles of the *EYS* gene and calculated the A/U nucleotide content. For c.4957dupA, A/U content was 67%, which is in the range previously reported (63%–71%). This result supports our hypothesis that transcripts having the frameshift mutation, c.4957dupA, may partially escape from NMD.

By analysis of *EYS* gene transcripts corresponding to exon 42–43, we unexpectedly found that the exon 42–43 region of the *EYS* gene is expressed in human dermal fibroblasts before photoreceptor-induction. Expression levels of this exon 42–43 fragment were decreased by photoreceptor-induction (Fig. 3, Panels **a** and **c**). These findings suggest that fibroblast-type transcripts exist. To confirm this hypothesis, endpoint RT-PCR was performed with primer pairs designed upstream and downstream of the predicted transcription start site of “fibroblast-type” transcripts (unpublished data, ARVO 5418–0070, 2018) (Fig. 3, Panel **d**). The conventional form expressed in the retina (NCBI, NM_001142800.1) was not expressed in fibroblasts, although the transcript was expressed in Y79. These results suggest that decrease of expression levels of the 42–43 fragments by photoreceptor-induction is derived from decrease of expression levels of “fibroblast-type” *EYS* gene transcripts. We have not yet determined how to discriminate the “fibroblast-type” from the conventional form. That will be done in a future study. The expression levels of the *EYS* gene fragment corresponding to exon 42–43 in Pt#2 having the nonsense mutation, c.8805C > A, were almost the same as those from normal volunteers and the amplified product had c.8805C > A, suggesting that transcripts with c.8805C > A may completely escape from degradation by NMD as expected, because this mutation produces the nonsense codon in the last exon [21].

Pt#3 has the truncating mutation in exon 8, c.1211dupA, discovered previously [5]. By endpoint RT-PCR and sequencing, transcripts derived from the other allele was only clearly detected and transcripts having c.1211dupA were extremely little (Fig. 4), suggesting that transcripts derived from the mutated allele disappeared by degradation by NMD as expected. Our results are also consistent with the findings of Nagy et al., that the PTC resides ≧ 50 nt upstream of an exon-exon junction stimulates NMD [33].

Our present study predicts that the frameshift mutation, c.4957dupA and the nonsense mutation, c.8805C > A, may produce truncated proteins, p.Ser1653Lysfs*2 and p.Tyr2935*, respectively. Although we tried Western blotting using commercially available antibodies against an N-terminal peptide of EYS, we could not detect a specific signal clearly at the expected size of around 300 kDa for cell lysates derived from photoreceptor-directed fibroblasts and Y79, as the authors of zebrafish models of EYS-RP claimed in a previous paper [38]. One of reasons could be that, because the EYS protein is a secreted protein, the EYS protein levels in tested cells may not have been high enough to be detected owing to secretion into extracellular space. Future experiments are required such as Western blotting using our original antibody against other N-terminal peptides of EYS.

In the present study, possible alternative aberrant transcripts of *EYS* were found between exon 26 and exon 27 during photoreceptor-directed differentiation (Fig. 5, Additional file 1: Figure S6). The variation of the *EYS* gene transcripts depending on alternative splicing have been reported; 43 exons (NCBI, NM_001142800), 44 exons (NCBI, NM_001292009), and 45 exons by Isackson et al. [39]. The significance of observed alternative splicing in the *EYS* gene is not clear so far, however, these sequences become a candidate for screenig new mutations by exome sequencing. To elucidate the molecular mechanism for phenotype variation of EYS-RP, we need to clarify alternative splicing variants of the *EYS* gene in detail.

The present study demonstrates evasion from NMD in *EYS* gene transcripts of EYS-RP patients with two kinds of truncating mutations. We also revealed the manner of decay of genetically defective *EYS* gene transcripts in photoreceptor-directed fibroblasts derived from EYS-RP depends on the type of mutation. It remains unclear whether this variation of decay manners of *EYS* gene transcripts is involved in variation of phenotype severity in EYS-RP patients because all three tested patients here were EYS-RP of almost the same severity. The present study also suggests that the redirect differentiation method could be a valuable tool for disease modeling despite some limitations.

## Conclusions

In summary, our study showed that expression levels of genetically defective *EYS* gene transcripts in photoreceptor-directed fibroblasts of EYS-RP patients vary depending on the type of mutation. Variation in expression levels in transcripts having c.1211dupA, c.4957dupA and c.8805C > A suggests that almost complete nonsense-mediated mRNA decay (NMD), partial NMD and escape from NMD occurred for these transcripts, respectively. The present study also suggests that the redirect differentiation method could be a valuable tool for disease modeling despite some limitations. To determine the relationship with phenotypic variations in EYS-RP patients, more samples are needed.

## Additional file

Additional file 1: **Table S1.** Primer sequences for RT-PCR. **Figure S1.** Cell growth of dermal fibroblasts with or without *EYS* gene defects. The number of cells increased until about 14 days; however, growth rates were different depending on the donor. The highest was N#1, followed in order by Pt#2, N#3 and Pt#1 (N#1>Pt#2>N#3≒Pt#1). Although the results suggest that the cell growth may be determined mainly by donor age and defects in the *EYS* gene are only relevant for younger EYS-RP patients, a larger sample size should be tested to assess differences in cell growth between EYS-RP patients and normal volunteers. **Figure S2.** Exogenous expression of transgenes, *CRX*, *RAX*, *NeuroD* and *OTX2*, in induced photoreceptor-like cells derived from fibroblasts of 3 arRP patients and 3 normal volunteers. Exogenous expression of 4 transgenes, *CRX*, *RAX*, *NeuroD* and *OTX2* initiated 10 days after gene transduction. **Figure S3.** Expression of CRX and blue opsin in induced photoreceptor-like cells derived from fibroblasts of 3 arRP patients and 3 normal volunteers. Immunocytochemistry using antibodies to CRX and blue opsin (green). **Figure S4.** Induction of retina-specific genes, blue opsin and S-antigen, in human dermal fibroblasts by the retroviral infection of genes for defined transcription factors. **Figure S5.** Expression levels of *EYS* gene transcripts over time during photoreceptor-directed differentiation by qRT-PCR. **a**. Changes of expression levels of *EYS* gene transcripts over time during photoreceptor-directed differentiation by qRT-PCR. Expression levels of the *EYS* gene in Pt#1, Pt#2 and N#2 reached maximum levels around 10 to 14 days after gene transduction and remained unchanged for up to 3 weeks. **b**. Statistical analysis of expression levels of *EYS* gene corresponding to exon 8-9 by qRT-PCR. Expression levels of exon 8-9 of Pt#1 and Pt#2 were almost the same as the average of three normal volunteers 10 days after gene transduction (Wilcoxon test, *p* = 0.1747 for Pt#1 and *p* = 0.1725 for Pt#2). **Figure S6.** The inserted sequence between exon 26 and exon 27. (PDF 846 kb)

## Abbreviations

arRP: autosomal recessive retinitis pigmentosa
EYS-RP: arRP with defects in the *EYS* gene
NMD: nonsense-mediated mRNA decay
NRCD: National Rehabilitation Center for Persons with Disabilities
PTC: premature termination codon
RP: retinitis pigmentosa
