# Supplementary material for: The manner of decay of genetically defective EYS gene transcripts in photoreceptor-directed fibroblasts derived from retinitis pigmentosa patients depends on the type of mutation
Source: Stem Cell Res Ther. 2018 Oct 25;9:279. doi: 10.1186/s13287-018-1016-9 (PMC6202841; doi:10.1186/s13287-018-1016-9)
Supplement: Supplementary file 1 — Table S1. Primer sequences for RT-PCR. Figure S1. Cell growth of dermal fibroblasts with or without EYS gene defects. The number of cells increased until about 14 days; however, growth rates were different depending on the donor. The highest was N#1, followed in order by Pt#2, N#3 and Pt#1 (N#1>Pt#2>N#3≒Pt#1). Although the results suggest that the cell growth may be determined mainly by donor age and defects in the EYS gene are only relevant for younger EYS-RP patients, a larger sample size should be tested to assess differences in cell growth between EYS-RP patients and normal volunteers. Figure S2. Exogenous expression of transgenes, CRX, RAX, NeuroD and OTX2, in induced photoreceptor-like cells derived from fibroblasts of 3 arRP patients and 3 normal volunteers. Exogenous expression of 4 transgenes, CRX, RAX, NeuroD and OTX2 initiated 10 days after gene transduction. Figure S3. Expression of CRX and blue opsin in induced photoreceptor-like cells derived from fibroblasts of 3 arRP patients and 3 normal volunteers. Immunocytochemistry using antibodies to CRX and blue opsin (green). Figure S4. Induction of retina-specific genes, blue opsin and S-antigen, in human dermal fibroblasts by the retroviral infection of genes for defined transcription factors. Figure S5. Expression levels of EYS gene transcripts over time during photoreceptor-directed differentiation by qRT-PCR. a. Changes of expression levels of EYS gene transcripts over time during photoreceptor-directed differentiation by qRT-PCR. Expression levels of the EYS gene in Pt#1, Pt#2 and N#2 reached maximum levels around 10 to 14 days after gene transduction and remained unchanged for up to 3 weeks. b. Statistical analysis of expression levels of EYS gene corresponding to exon 8-9 by qRT-PCR. Expression levels of exon 8-9 of Pt#1 and Pt#2 were almost the same as the average of three normal volunteers 10 days after gene transduction (Wilcoxon test, p = 0.1747 for Pt#1 and p = 0.1725 for Pt#2). Figure S6. The in [file 13287_2018_1016_MOESM1_ESM.pdf]

Table S1 Primer sequences for RT-PCR

Primer Name	Sequences (5'-3')
EYS Ex 4-F	CCAATTCCCAGGAATCCTTAACCACAAC
EYS Ex 11-R	CCAGATACATGTTGCCAGCCCATCTGAG
EYS Ex 10-58F	ACTGCACTGAAGATGCAACCTATG
EYS Ex 13-657R	GGCAGAAGTAATTACCAGGTTGGTC
EYS Ex 25-4F	TGCTCCATTGGGCTTCTTTGTGG
EYS Ex 26-598R	GACATCGAGGGGCTGAGCAATC
EYS Ex 26-4504F	GCTTCAAGCAACCAGAGACTCAC
EYS Ex 27-5774R	TGCTTGACATACAGCAGAAGTCC
EYS Ex 42-8093F	CTTTCAGAAGCAATGAGTTATCCTG
EYS Ex 43-9096R	CATAGGCACAGAGATTCTTTCTCC
EYS Ex 6-873F	TGAGGTGTCAGCAAAACCTTGTG
EYS Ex 11-1678R	CAGCCCATCTGAGAAAACATAGATACC
qEYS Ex 8-1236F	TAAACTGCTCAGCATCAACTGTC
qEYS Ex 9-1424R	GCAGGACCTTTATCTTGGCAA
EYS Ex 36-7140F	TGGAAATGGTGCCACCTGTGTTC
EYS Ex 37-7286F	TCCTGGCTTATTCACGGATCTCAGAC
EYS Ex 41-7945R	CTGTCTCTGTGCAGAATGATCCTTTCC
EYS Ex 40-7535R	TGGAAATGGTGCCACCTGTGTTC
EYS Ex 40-7818R	AACACTGCGTCCAGCATTTGGG
qEYS Ex 24-3640F	GAGAATGAACCTGGCTCGAC
qEYS Ex25-3818R	AAGCTGCTGACCAAAGTCTC
qEYS Ex42-8177F	CTCTCGCTGCAGATGGTATCC
qEYS Ex43-8293R	CGCCAAGGTTGTAGCGAAGT

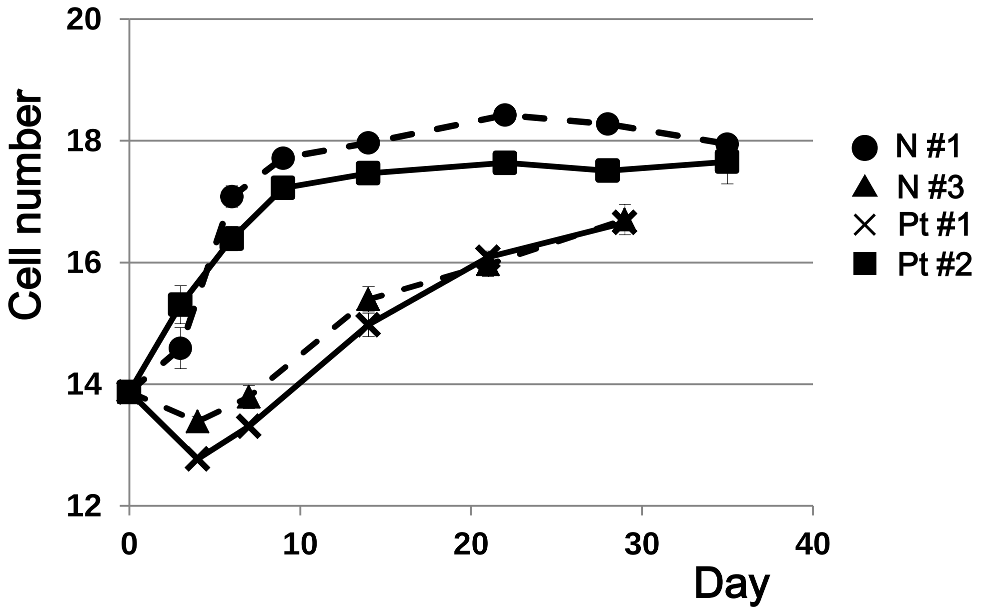

Figure S1: Cell growth of dermal fibroblasts with or without *EYS* gene defects. Vertical axis indicates Log2 (cell number) (mean ± SE, n=3) and horizontal axis indicates days after seeding. “Cell number” means “total number of adherent cells in a 12-well culture dish”. The number of cells at day 0 is that of seeded cells. We investigated the growth of dermal fibroblasts of N#1 (53 years old), N#3 (63 years old), Pt#1 (67 years old) and Pt#2 (58 years old). The number of cells increased until about 14 days; however, growth rates were different depending on the donor. The highest was N#1, followed in order by Pt#2, N#3 and Pt#1 (N#1>Pt#2>N#3≒Pt#1). The representative data from 3 independent experiments are shown. Although the results suggest that the cell growth may be determined mainly by donor age and defects in the *EYS* gene are only relevant for younger EYS-RP patients, a larger sample size should be tested to assess differences in cell growth between EYS-RP patients and normal volunteers.

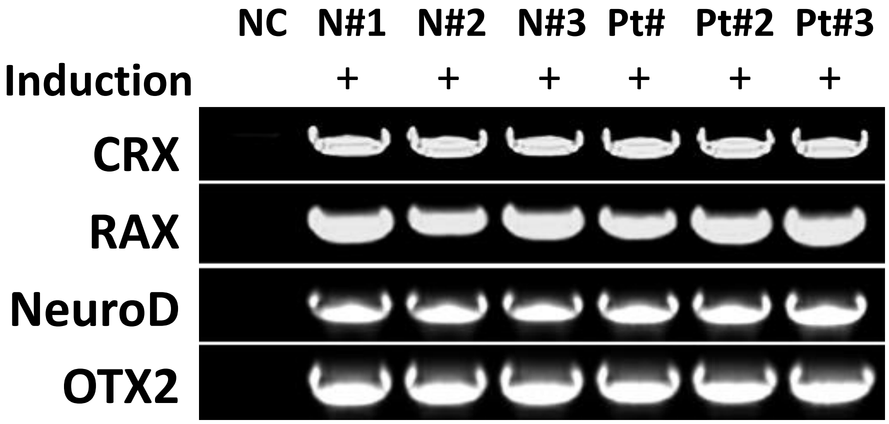

Figure S2: Exogenous expression of transgenes, *CRX*, *RAX*, *NeuroD* and *OTX2*, in induced photoreceptor-like cells derived from fibroblasts of 3 arRP patients and 3 normal volunteers.

RT-PCR analysis for 4 transgenes, *CRX*, *RAX*, *NeuroD* and *OTX2*. In human dermal fibroblasts of 3 normal volunteers (N#1, N#2, N#3) and 3 arRP patients (Pt#1, Pt#2, Pt#3), exogenous expression of 4 transgenes, *CRX*, *RAX*, *NeuroD* and *OTX2* initiated 10 days after gene transduction. The PCR primers are shown in our previous paper [17]. Only exogenous expression of transgenes was detected because the forward primer is from pMXs-DEST.

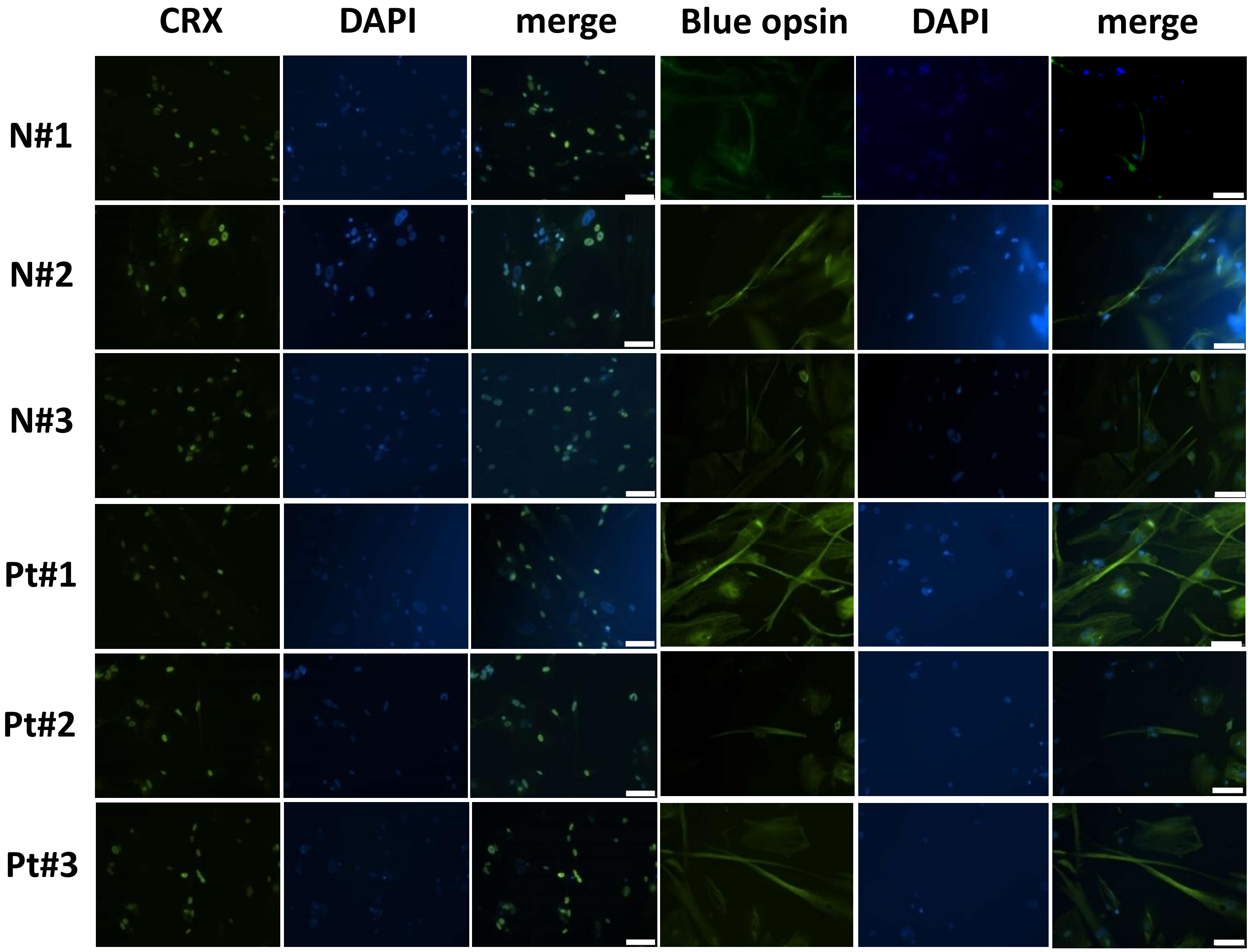

Figure S3: Expression of CRX and blue opsin in induced photoreceptor-like cells derived from fibroblasts of 3 arRP patients (Pt#1, Pt#2, Pt#3) and 3 normal volunteers (N#1, N#2, N#3).
Immunocytochemistry using antibodies to CRX and blue opsin (green). Nuclei were stained with DAPI (blue). Experiments were performed 14 days after transduction of *CRX*, *RAX*, *NeuroD* and *OTX2*. Scale bars represent 50 µm.

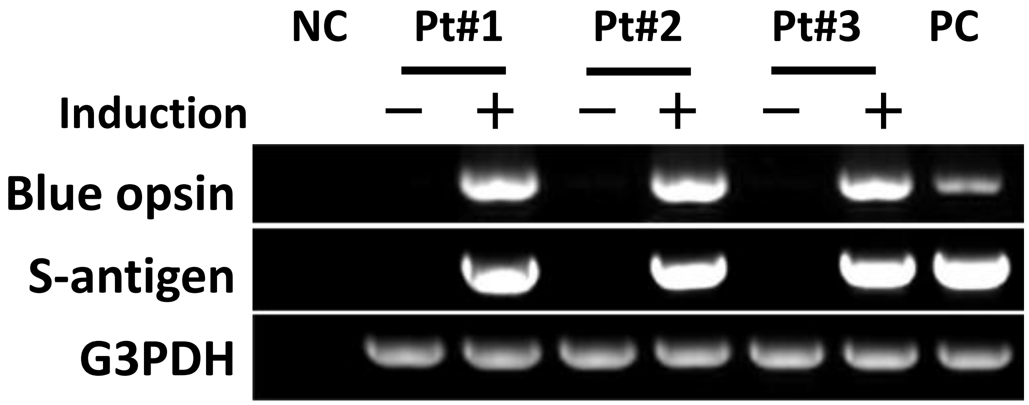

Figure S4: Induction of retina-specific genes in human dermal fibroblasts by the retroviral infection of genes for defined transcription factors.
RT-PCR analysis for photoreceptor-specific genes, blue opsin and S-antigen.
In human dermal fibroblasts of EYS-RP patients (Pt#1, Pt#2, Pt#3), blue opsin and S-antigen were induced by *CRX*, *RAX*, *NeuroD* and *OTX2* 10 days after gene transduction. "PC": a human retinal tissue as a positive control in 1:100 dilution compared to other samples. "NC": water as a negative control.

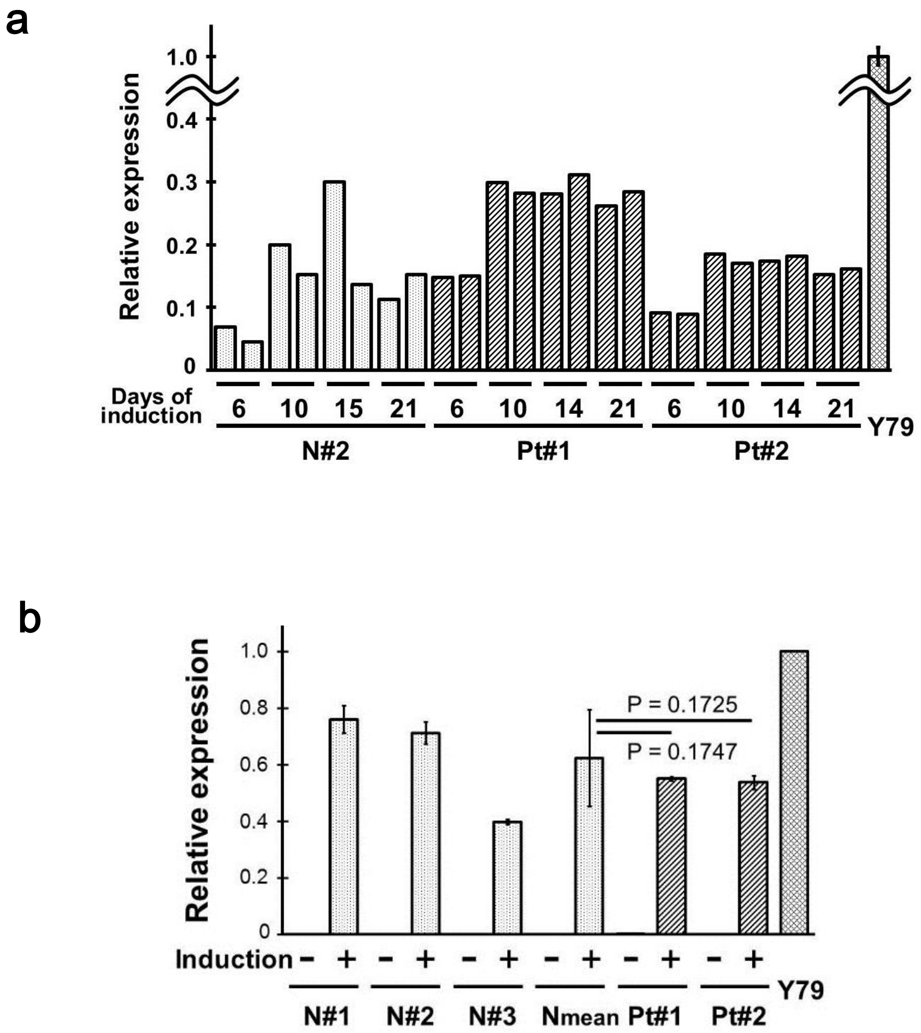

Figure S5: Expression levels of *EYS* gene transcripts over time during photoreceptor-directed differentiation by qRT-PCR.

(a) Changes of expression levels of *EYS* gene transcripts over time during photoreceptor-directed differentiation by qRT-PCR.

Vertical axis indicates relative expression (RQ calculated by built-in software with StepOnePlus™ (ThermoFischer)). Cells cultured in duplicate in 6-well dishes were counted 2 hours (day 0), 7, 14, 21, 28 and 35 days after seeding. Expression levels of the EYS gene in Pt#1, Pt#2 and N#2 reached maximum levels around 14 days after gene transduction and remained unchanged for up to 3 weeks. qRT-PCR was performed using primer pairs corresponding to exon 8-9. All of cDNA samples were 1:10 diluted for the EYS gene and 1:50 diluted for G3PDH.

(b) Analysis of expression levels of *EYS* gene corresponding to exon 8-9 by qRT-PCR.

Vertical axis indicates relative expression (mean ± SD, n=4 for Pt#1 and Pt#2, n=3 for N#2). Expression levels of exon 8-9 of Pt#1 and Pt#2 were almost the same as the average of three normal volunteers 10 days after gene transduction. cDNA samples of Y79 was 1:10 diluted for the EYS gene and 1:50 for G3PDH. cDNA derived from photoreceptor-directed fibroblasts were used in undiluted solution for the EYS gene and were 1:50 diluted for G3PDH.

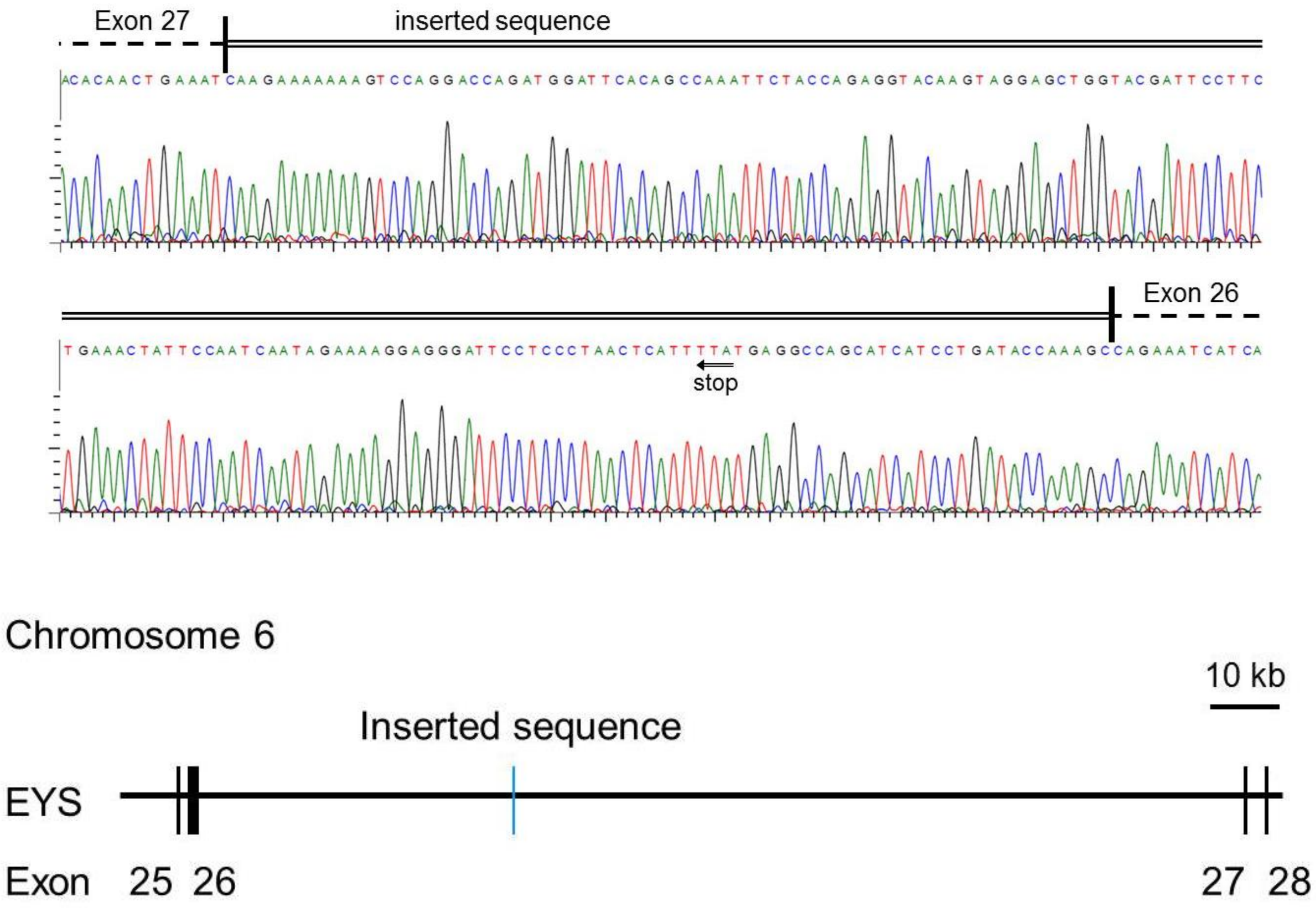

Figure S6: The inserted sequence between exon 26 and exon 27.
Upper panel is the inserted sequence between exon 26 and exon 27 detected in RT-PCR products with a different mobility (Fig. 5, panel B). The length of this inserted sequence is 161 nt and corresponds to 65254831-65254941 in Chr. 6 (Lower panel), which is predicted to be made by alternative splicing as a new exon.
